# Assessing the nonhuman primate reservoir of *Schistosoma mansoni* in Africa: a systematic review

**DOI:** 10.1186/s40249-019-0543-7

**Published:** 2019-05-10

**Authors:** Lindsay Richards, Berhanu Erko, Keerati Ponpetch, Sadie J. Ryan, Song Liang

**Affiliations:** 1 Department of Microbiology & Cell Science, University of Florida, Gainesville, FL 32610 USA; 2 Aklilu Lemma Institute of Pathobiology, Addis Ababa University, Addis Ababa, Ethiopia; 3 Department of Environmental and Global Health, College of Public Health and Health Professions, University of Florida, Gainesville, FL 32610 USA; 4 Department of Geography, University of Florida, Gainesville, FL 326102 USA; 5 Emerging Pathogens Institute, University of Florida, Gainesville, FL 32610 USA

**Keywords:** *Schistosoma mansoni*, Nonhuman primate, Infection, Transmission, Systematic review, Africa

## Abstract

**Background:**

Reports of natural infections of *Schistosoma mansoni* in a number of species of nonhuman primates (NHPs) in Africa, coupled with the substantial overlap of NHP habitats and human schistosomiasis endemic areas, has led to concerns about the role of NHPs in the transmission of human schistosomiasis. We conducted a systematic review of the literature to describe the current scope of knowledge for Africa, for the NHP species implicated, their geographical distribution, infection rates with *S. mansoni*, and to discuss the implications for public health and conservation*.*

**Main text:**

A systematic search of the literature was performed using PubMed, Web of Science, Google Scholar, the World Health Organization (WHO) library database, World Cat, and ScienceDirect without any language restriction. Studies examining *S. mansoni* infection of any African NHP species were included. Study types, primate species, their geographical distribution, and parasite diagnostic techniques reported in the studies were qualitatively summarized. Data for species with sample sizes ≥10 were included in the meta-analysis. We assessed the reported infection rate, and used a random-effects model to estimate the summary infection rates and 95% confidence intervals (*CI*s). We assessed heterogeneity among studies using the *I*^2^ statistics. Twenty-nine publications, from 1960 to 2018, were identified and included in the review. The studies examined a total of 2962 primates belonging to 22 species in 11 genera across ten countries (Cameroon, Eritrea, Ethiopia, Gabon, Kenya, Nigeria, Senegal, Tanzania, Uganda, and Zimbabwe), and *S. mansoni* infections were found in nine species of five genera in all countries. When we excluded studies with sample sizes < 10, data from 24 studies on 11 species of primates in three genera in ten countries remained in the meta-analysis. The overall pooled estimate of infection rate was 10% (95% *CI*: 6–16%) with high heterogeneity (*I*^2^ = 94.77%) across countries and species/genera. Among the three genera, *Pan* had the highest infection rate of 15% (95% *CI*: 0–55%), followed by *Papio* at 11% (95% *CI*: 6–18%), and *Cercopithecus* at 5% (95% *CI*: 0–14%). The association between NHP and human infections was positive, but not significant, due to low study sample matches and high variation.

**Conclusions:**

Our findings suggest that *S. mansoni* infection rate is high in African NHPs, with substantial heterogeneities across species/genera and countries in Africa. Given the evidence for potential spillover and spillback of *S. mansoni* between African NHPs and humans, further research is urgently needed to understand ecology and mechanisms of transmission of the parasite between NHP and human hosts, in order to inform control strategies of this important neglected tropical disease.

**Electronic supplementary material:**

The online version of this article (10.1186/s40249-019-0543-7) contains supplementary material, which is available to authorized users.

## Multilingual abstracts

Please see Additional file 1 for translations of the abstract into the five official working languages of the United Nations.

## Background

Schistosomiasis is one of the most important neglected tropical diseases (NTDs) in the world, with almost 800 million people at risk and more than 240 million people infected in 78 countries, causing high disease burden in many endemic areas, in particular in Sub-Saharan Africa [1–3]. There are five major schistosome species that infect humans: namely *Schistosoma mansoni, S. haematobium, S. japonicum, S. intercalatum*, and *S. mekongi.* The majority of human infections occur in Africa, caused by *S. mansoni* and *S. haematobium*, whereas *S. japonicum* and *S. mekongi* are confined to Asia. *S. mansoni* is also found in South and Central America [4–8]. Schistosomiasis is prevalent in tropical and subtropical regions, and transmission is associated with socio-environmental risk factors such as lack of access to safe water and appropriate sanitation, age- and occupation-related exposure, environmental changes (e.g., related to water development projects, land-use, and climate change), and human susceptibility to infection [3, 9–12].

The transmission of some human *Schistosoma* species, in particular for *S. japonicum, S. mekongi,* and *S. mansoni*, involves nonhuman mammalian hosts. The former two are widely recognized zoonoses that can infect a wide range of mammalian hosts [13, 14]. Natural infections of *S. japonicum* have been reported in more than 40 species of wild and domestic mammals, some of which have played important roles in maintaining the human schistosomiasis transmission cycle [14–17]. For example, studies in Poyang Lake, China, suggested that bovids, particularly water buffaloes (*Bubalus bubalis*), were key reservoirs of *S. japonicum,* contributing up to 75% of human infections in the region [18, 19], and similar findings were reported in the Philippines [20–22]. *S. mansoni*, which is primarily distributed across Africa, the Middle East, and parts of the Americas, has zoonotic potential due to its prevalence in nonhuman primates (NHPs), but there is a lack of conclusive information about its role as a zoonosis.

*S. mansoni*, the species of interest for this review, causes intestinal schistosomiasis. The principal intermediate hosts for *S. mansoni* are freshwater snails of the genus *Biomphalaria*. Infected snails shed cercariae, the larval, infectious form of the parasite, into the aquatic environment, where they then penetrate the skin of definitive hosts (e.g. humans) upon contact with contaminated water. Once inside the definitive hosts, the larvae develop into adults, which live in the blood vessels, where they mate and release eggs. These eggs can then be passed via the host’s faeces into freshwater. These then hatch out, as miracidia, which in turn infect the snail hosts, completing the parasite transmission lifecycle. *S. mansoni* can infect both humans and NHPs, suggesting that when NHPs live in proximity to human communities, the two may share the endemic environment, creating opportunities for parasite spillover and local transmission maintenance.

Natural infections of *S. mansoni* in many species of NHPs have been reported, but the epidemiological role of NHPs in transmission, in particular their contribution to human infections, remains largely unknown. This is in part due to a dearth of information on interactions (e.g. exposure to the parasite and contamination of the environment) between humans and NHPs in the shared endemic environment, and an incomplete understanding of the transmission dynamics across the hosts. As the first step in establishing baseline information to inform and identify avenues for future research, we conducted a systematic review of *S. mansoni* infections in NHPs in Africa, to summarize information on NHP species implicated, *Schistosoma* infection status in African NHPs, their geographical distributions, and potential implications for human infection, based on the available evidence.

## Main text

### Search strategy and selection criteria

We conducted a comprehensive search for literature on *S. mansoni* infections among NHPs in Africa. This was done by searching databases including PubMed, Google Scholar, Web of Science, WHO library database, World Cat, and ScienceDirect, using the keywords “*Schistosoma mansoni*”, “*S. mansoni*”, “schistosomiasis”, “schistosome”, “zoonosis”, “primate”, “helminth”, and “Africa”. No time frame was specified in the search, but all of the studies identified occurred in the 1960s or later. The search was repeated in PubMed with no language constraint, but no additional results were returned. The reference lists of sources identified in this search were examined for additional relevant studies. Books, dissertations, conference abstracts, and unpublished reports were included.

Search results were initially screened based on title and abstract to determine eligibility. Those that mentioned *S. mansoni* and primates and took place in Africa were then further examined for relevant content. Each study needed to meet the following inclusion criteria: reporting *S. mansoni* infections in NHPs; occurring in Africa; and reporting natural infections rather than induced (e.g., laboratory-based infection).

### Data extraction and outcome measure

Upon identification of eligible studies, relevant information from each study including survey location (e.g., site and country), year, primate species and genus, type of survey, diagnostic test, number of primates surveyed, and outcome measured (e.g., number of positive and negative tests) were extracted and entered into a standardized spreadsheet. Coordinates of the survey’s location were identified from a Google search of the recorded study site. Type of survey described whether the primates were free-ranging or captive, and diagnostic procedure was based on parasitological tests (e.g., using faecal samples), serological tests (e.g., circulating antigen of the parasite), tissue examination, or necropsy. To explore whether there was an association between NHP and human infections, for each eligible study, we also searched for studies for corresponding human data based on a spatiotemporal ‘match’ of geographical area (e.g. at the scale of district or smaller) and survey year (e.g. ± 5 years).

### Statistical analysis and mapping

For all included studies, a qualitative summary of key study characteristics is provided. Data on the outcome measure (e.g., infection rate) were grouped by country and primate genus and were included in the meta-analysis using a random-effect model. In the analysis, various diagnostic tests were not differentiated. While heterogeneity statistics were estimated in the model for all studies (overall), studies within group, and between group comparison, *I*^2^ (e.g., measuring the percentage of variations due to inter-study heterogeneity) and associated *P-*values [23] were reported for the former two for comparisons including four or more studies, and only *P-*value was reported for between group comparison. The distribution of studies, primate genera, and infection level were mapped for qualitative comparisons. Statistical analysis was performed in Stata (V11.1, StataCorp, College Station, Texas, USA) and mapping was conducted in ArcGIS (V10.2.2, ESRI, Redlands, California, USA).

## Results

**Fig. 1:**
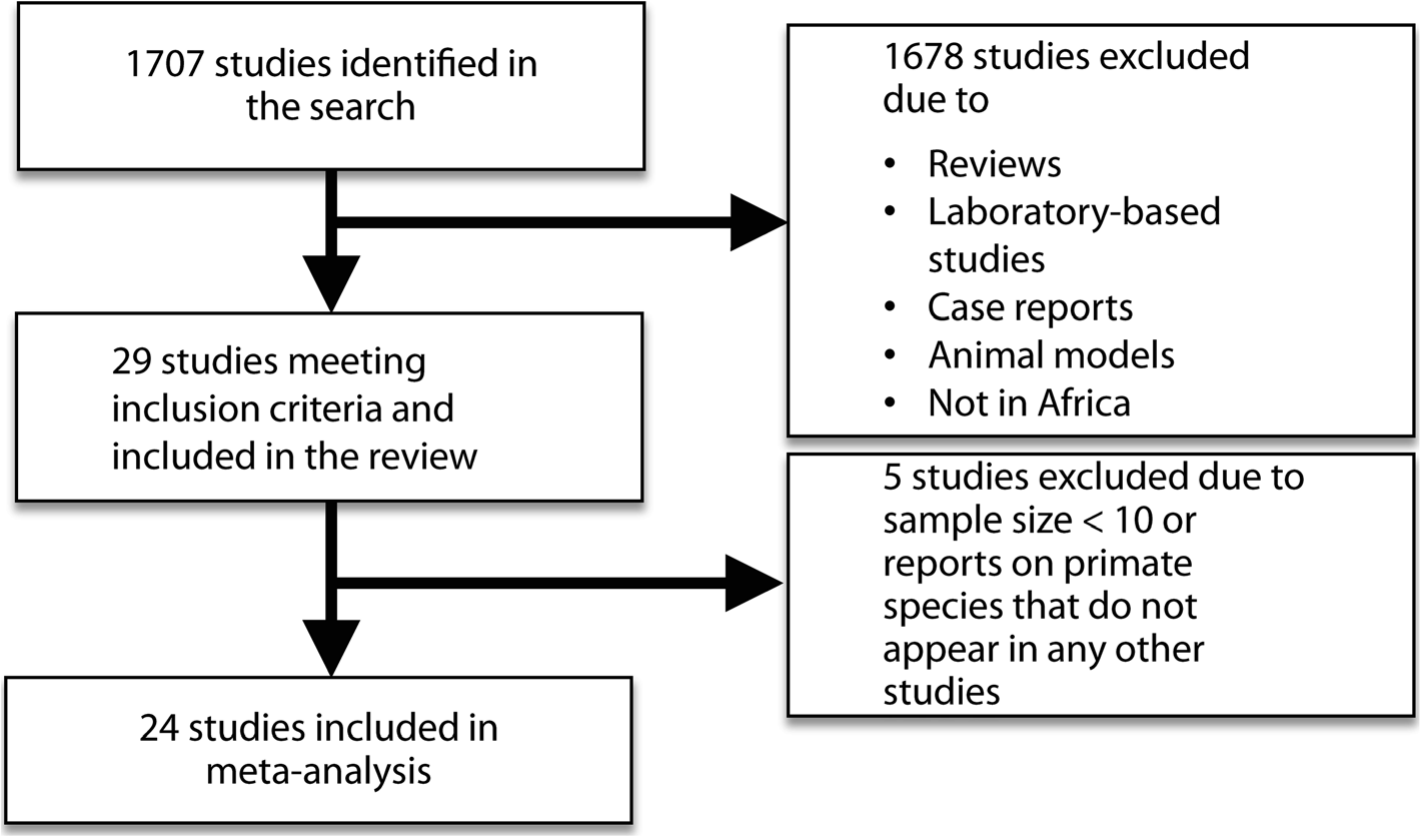
Flowchart showing inclusion and exclusion of studies on *Schistosoma mansoni* infections of nonhuman primates in Africa and search results

**Fig. 2:**
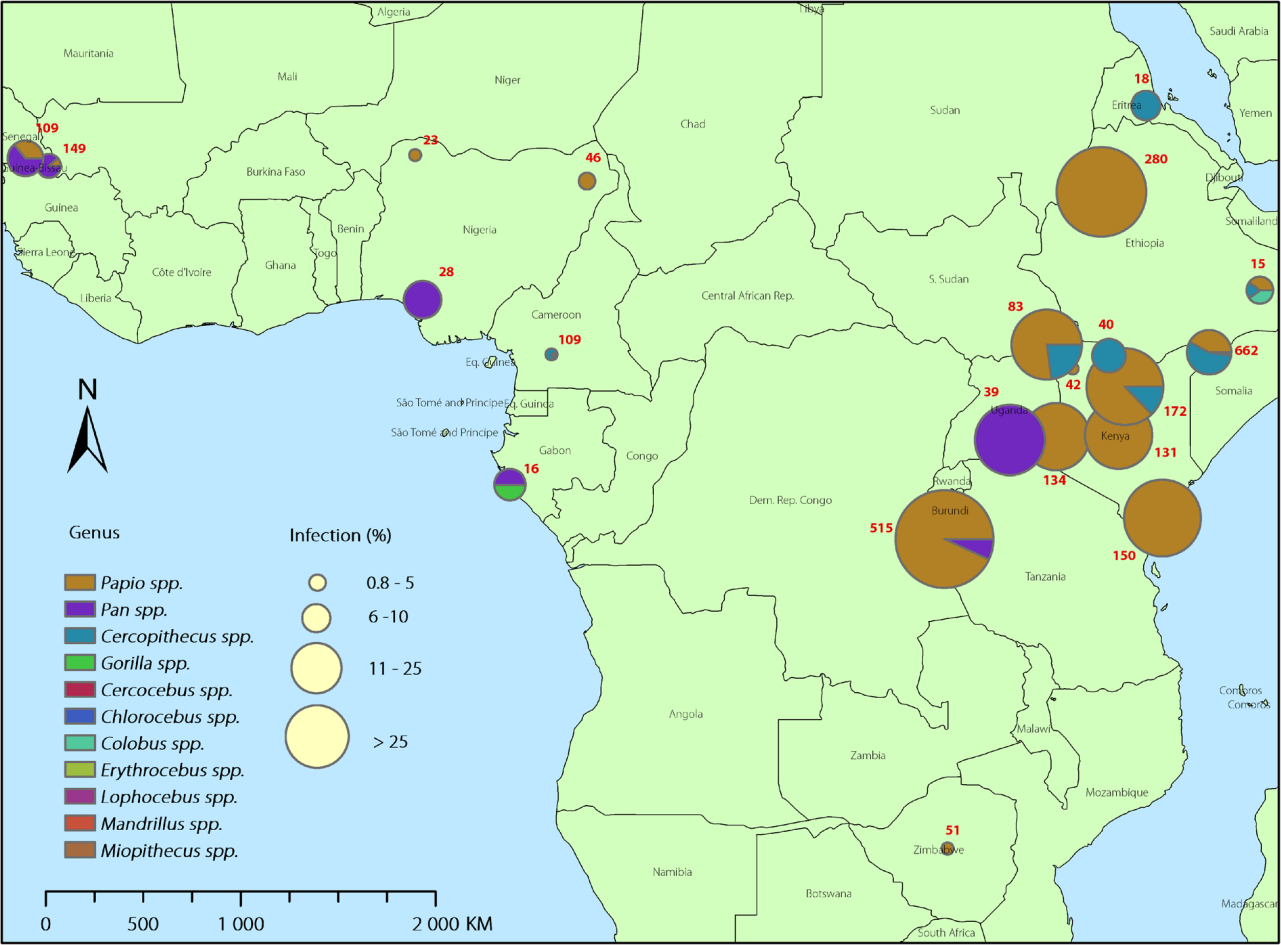
Distribution of included studies and reported *Schistosoma mansoni* infections of nonhuman primates in Africa

The search process and results are shown in Fig. 1. In total, 29 studies from 1960 to 2018 were included, and key characteristics of the studies summarized in Table 1. The studies encompassed ten countries, including Cameroon, Eritrea, Ethiopia, Gabon, Kenya, Nigeria, Senegal, Tanzania, Uganda, and Zimbabwe, exhibiting a wide geographical distribution across Africa (Fig. 2) and substantial variation in sample size (ranging from three to 206 by species) and infection rate (ranging from zero up to 90%) among primate species across the regions (Table 1). A total of 22 species of NHPs, belonging to ten genera, were represented in the studies. There were 69 species-specific surveys, among which, 39 reported one or more infected primates. Among the 29 studies, 24 with sample size > 10 were included in the meta-analysis. The 24 studies included primates from all ten countries, seven species (i.e. *Cercocebus torquatus, Cercopithecus aethiops, Cercopithecus mitis, Pan troglodytes, Papio anubis, Papio cyanocephalus,* and *Papio hamadryas*), belonging to three genera (i.e. *Cercopithecus, Pan,* and *Papio*). Overall, high heterogeneities in infection rate were observed across the NHP species/genera and survey sites/country (Table 1). Due to small sample numbers for some species and sites, our meta-analysis was performed at the scales of genus and country. The country with the highest rate of *S. mansoni* infections in NHPs was Eritrea, with an infection rate of 33% (95% *CI*: 16–56%) reported in one study that took place in 1970.

**Table 1:** Key characteristics of included studies on *Schistosoma mansoni* infections in nonhuman primates in Africa

Year	Site	Host species	Study type	Diagnostic technique	% (infected/ examined)	Reference
1960	Kenya: Athi & Kibwezi Rivers	*Papio anubis*	Wild	Necropsy	23.88 (32/134)	Miller [24]
1960	Kenya:Machakos District and Lake Albert	*Papio anubis*	Wild	Necropsy	54.69 (35/64)	Nelson [25]
1961	Kenya	*Papio anubis*	Wild	Necropsy	28.00 (42/150)	Strong et al. [26]
1969	Tanzania: Lake Manyara National Park	*Papio anubis*	Wild	Necropsy	80.00 (4/5)	Fenwick [27]
1970	Eritrea: Asmara	*Cercopithecus aethiops*	Wild	FECT	33.33 (6/18)	Cheever [28]
1970	Tanzania: Lake Victoria	*Cercopithecus aethiops*	Wild	FECT	23.40 (11/47)	Cheever [28]
1972	Tanzania	*Papio spp.*	Wild	Necropsy	16.67 (2/12)	Taylor et al. [29]
1974	Zimbabwe	*Papio ursinus*	Wild	Necropsy	1.96 (1/51)	Goldsmid [30]
1979	Ethiopia: Omo National Park	*Cercopithecus aethiops* *Papio anubis*	Wild	Necropsy	CA: 66.67 (2/3) PA: 50.00 (3/6)	Fuller et al. [31]
1982	Kenya: Rift Valley, Lake Naivasha	*Cercopithecus aethiops*	Wild	Kato-Katz	20.00 (8/40)	Else et al. [32]
1989	Senegal: Mt. Assirik	*Papio papio*	Wild	FECT	23.01 (9/39)	McGrew et al. [33]
1992	Kenya: Kibwezi, Kivungoni Lake	*Papio anubis*	Wild	FECT	24.43 (32/131)	Muchemi [34]
1997	Tanzania: Gombe National Park	*Papio cynocephalus*	Wild	FECT	18.93 (39/206)	Muller-Graf et al. [35]
1998	Kenya: Nairobi	*Papio cynocephalus*	Wild	Kato-Katz	4.35 (4/92)	Munene et al. [36]
1998	Kenya: Nairobi	*Papio cynocephalus* *Cercopithecus aethiops*	Wild	FECT	PC: 3.60 (4/111) CA: 4.88 (6/123)	Muriuki et al. [37]
1999	Tanzania: Gombe National Park	*Papio cynocephalus*	Wild	ZSF	2.86 (1/35)	Murray et al. [38]
2001	Ethiopia: Bishan Gari; Ethiopia: Burka Dita	*Papio anubis*	Wild	Kato-Katz	BG: 26.15 (34/130) BD: 12.09 (11/91)	Erko et al. [39]
2003	Kenya: Mpala Wildlife Research Centre	*Papio cynocephalus*	Wild	FECT	2.38 (1/42)	Hahn et al. [40]
2004	Ethiopia: Bishan Gari; Ethiopia: Burka Dita	*Papio anubis*	Wild	FECT	BG: 33.33 (8/24) BD: 11.43 (4/35)	Legesse et al. [41]
2006	Nigeria: Calabar, Afi Mountain Primate Conservation Area	*Papio anubis*	Wild	FECT	4.35 (1/23)	Weyher et al. [42]
2011	Senegal: Fongoli	*Papio hamadryas*	Wild	SNF & FECT	23.53 (4/17)	Howells et al. [43]
2011	Nigeria: Calabar, Afi Mountain Primate Conservation Area	*Pan troglodytes*	Semi-captive	MMT & MBT	35.71 (10/28)	Mbaya & Udendeye [44]
2011	Cameroon: Yaounde	*Erythrocebus patas*	Captive	FECT	33.33 (1/3)	Pourrut et al. [45]
2011	Uganda: Lake Victoria, Ngamba Island	*Pan troglodytes*	Wild	ELISA, CCA, Kato-Katz, qPCR	89.74 (35/39)	Standley et al. [46]
2012	Tanzania: Gombe National Park	*Papio anubis*	Wild/ habituated	FECT	11.90 (15/126)	Bakuza [47]
2013	Nigeria: Yankari National Park	*Papio anubis*	Wild	FECT	4.30 (2/46)	Mafuyai et al. [48]
2016	Gabon: Loango National Park	*Gorilla gorilla* *Pan troglodytes*	Wild	MSF, MIF	GG: 62.50 (5/8) PT: 25.00 (2/8)	Cervena et al. [49]
2017	Kenya: Tsavo West National Park	*Papio anubis.*	Semi- captive	FECT	2.13 (1/47)	Chimoyi [50]
2018	Ethiopia: Oromia Regional State	*Cercopithecus aethiops* *Papio anubis*	Wild	Kato-Katz	CA: 21.60 (8/37) PA: 50.72 (35/69)	Kebede et al. [51]

Necropsy: Autopsy; *FECT* Formal-Ether Concentration Technique, *Kato-Katz* Kato-Katz thick smear, *ZSF* Zinc Sulfate Flotation, *SNF* Sodium Nitrate Flotation, *ELISA* Enzyme-Linked Immunosorbent Assay, *CCA* Circulating Cathodic Antigen Test, *qPCR* Real-time Polymerase Chain Reaction, *MSF* Modified Sheather’s Flotation, *MIF* Merthiolate-Iodine-Formalin Fecal Technique, *MMT* Modified McMaster Technique, *MBT* Modified Baerman’s TechniqueIf studies did not include a specific site, only the country is provided in this table

**Fig. 3:**
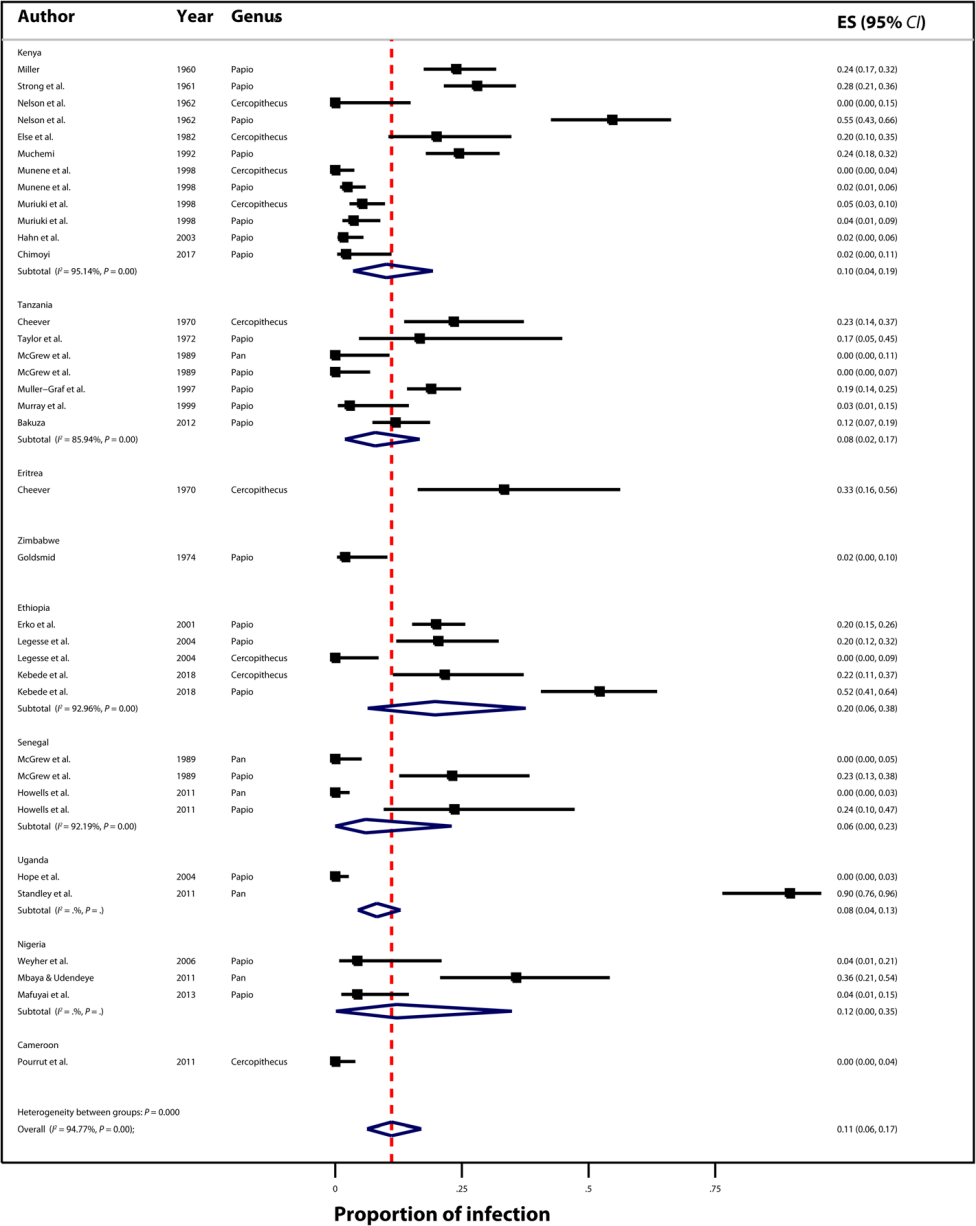
Forest plot and pooled estimates of nonhuman primate *Schistosoma mansoni* infections by country (ES: Effect size)

Overall, the analysis shows significant heterogeneity (*I*^2^ = 94.77%, *P* = 0.00) across all studies, studies within country (Kenya (*I*^2^ = 95.14%, *P =* 0.00), Tanzania (*I*^2^ = 85.94%, *P* = 0.00), Ethiopia (*I*^2^ = 92.96%, *P =* 0.00), and Senegal (*I*^2^ = 92.19%, *P =* 0.00)), no *I*^2^ estimates for other countries due to limited studies (Fig. 3), and significant heterogeneity between groups (*P =* 0.00). In Ethiopia, three studies (2001–2018) on *Cercopithecus* and *Papio* (*Cercopithecus aethiops* and *Papio anubis*) reported infection rates ranging from 0 to 52%, with a pooled estimate of infection rate of 20% (95% *CI*: 6–38%). In Nigeria, three studies (2006–2013) on *Papio* and *Pan* (*Papio anubis* and *Pan troglodytes*) reported infection rates ranging from 4 to 36%, with a pooled estimate of infection of 12% (95% CI: 0–35%). In Kenya where nine studies (1960–2017) examined four species (*Cercopithecus aethiops, Cercopithecus mitis, Papio anubis,* and *Papio cyanocephalus*), reported infection rates ranged between 0 and 55%, with a pooled estimate of infection rate at 10% (95% *CI*: 4–19%). In Tanzania, six studies (1970–2012) on four species (*Cercopithecus aethiops, Pan troglodytes, Papio anubis,* and *Papio cyanocephalus*) reported infection rates between 0 and 23%, and had a pooled estimate of 8% (95% *CI*: 2–17%). In Uganda, the pooled estimate of infection rates was 8% (95% *CI*: 4–13%) based on two studies on two species (*Pan troglodytes* and *Papio cyanocephalus*), and in Senegal, the pooled estimate of infection rates was 6% (95% *CI*: 0–23%) based on two studies on three species (*Pan troglodytes*, *Papio hamadryas,* and *Papio papio*) (Fig. 3).

**Fig. 4:**
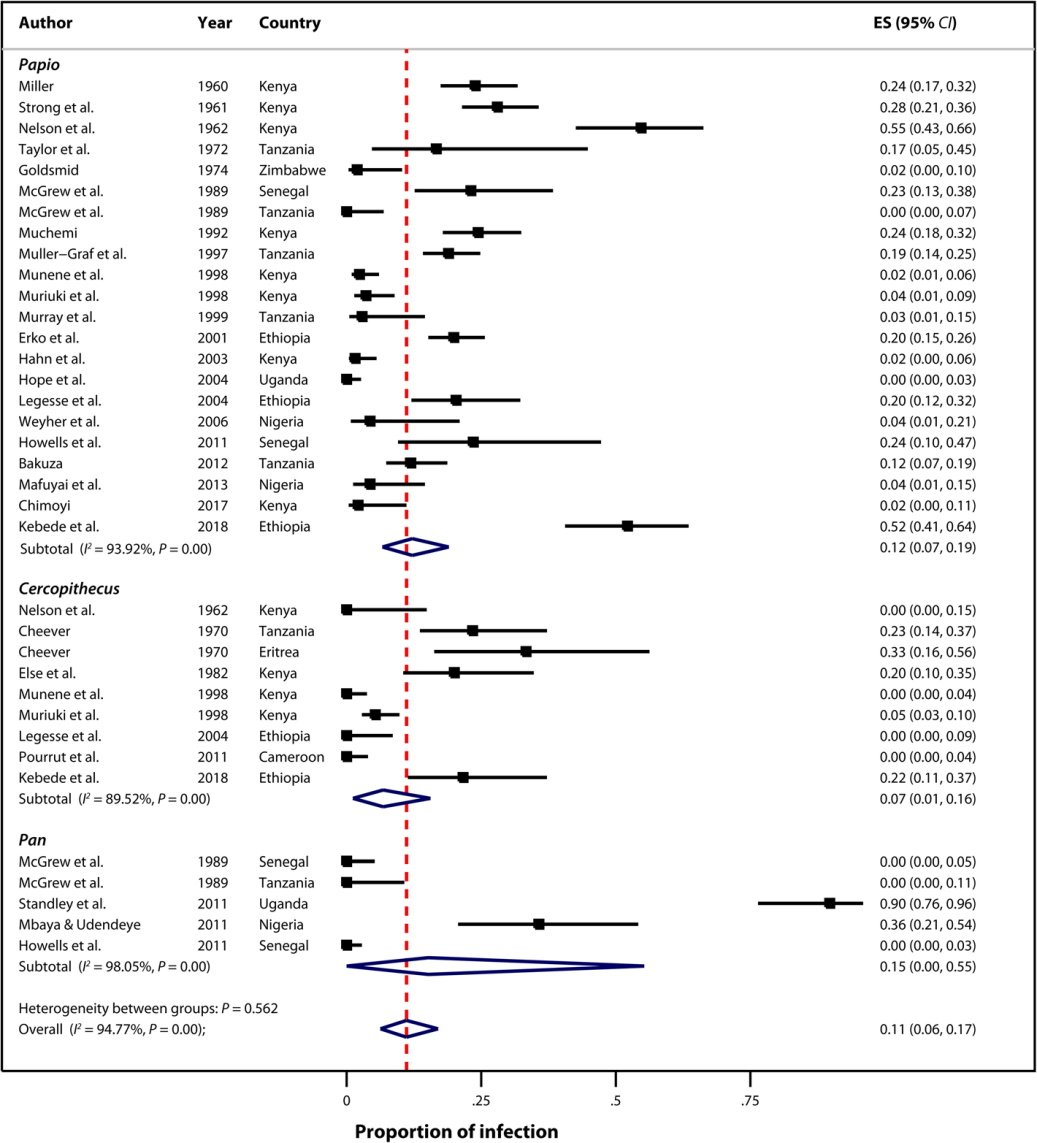
Forest plot and pooled estimates of nonhuman primate *Schistosoma mansoni* infections by genus (ES: Effect size)

For analysis by genus, the finding shows significant heterogeneity in studies within NHP genus group (*Papio* [*I*^2^ = 93.92%, *P = 0.00*], *Cercopithecus* [*I*^2^ = 89.52%, *P =* 0.00], and *Pan* [I^2^ = 98.05%, *P =* 0.00]) but not between  groups (*P = 0.562*) (Fig. 4). One species (*Pan troglodytes*) of primate in the genus *Pan* was reported in four studies spanning from 1989 to 2011 with a pooled estimate of infection rate of 15% (95% *CI*: 0–55%). For the genus *Papio*, five species (*Papio anubis, Papio cyanocephalus, Papio hamadryas, Papio papio,* and *Papio ursinus*) were reported in 21 studies from 1960 to 2018 with a pooled estimate of infection rate of 12% (95% *CI*: 6–19%). For the genus *Cercopithecus*, two species (*Cercopithecus aethiops* and *Cercopithecus mitis*) were reported in seven studies spanning from 1962 to 2018 with a pooled estimate of infection rate of 7% (95% *CI*: 1–16%) (Fig. 4). The majority of studies (27) examined wild NHPs while two examined captive NHPs [36, 46] . Most studies diagnosed infection using fecal samples, and other methods included serological tests and necropsy (Table 1).

**Fig. 5:**
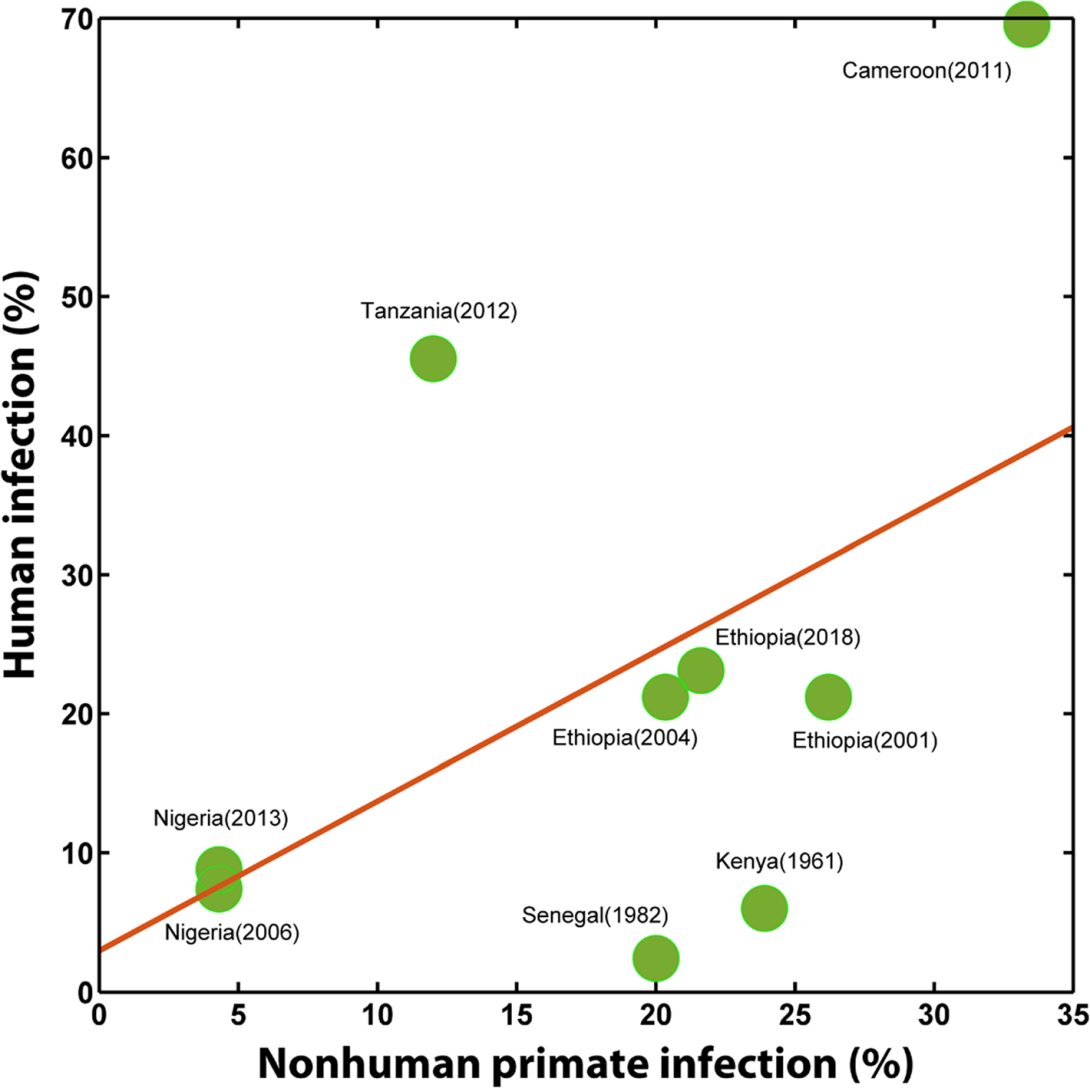
Association between nonhuman primate and human infections by *Schistosoma mansoni*

Nine studies were found to have a corresponding spatiotemporal ‘match’ of human schistosomiasis infection data based on site and survey year. Overall the association between human and nonhuman infections tended to be positive, although low sample size and high variation precluded statistical significance (Fig. 5, Pearson’s *R* = 0.48, *P* = 0.19).

## Discussion

Over the past several decades, extensive efforts to control schistosomiasis have transformed global patterns of the disease, motivating the development of an agenda to achieve global elimination of schistosomiasis as a public health problem in the years to come [3]. Among different control strategies in current use, controlling infection sources from nonhuman animal reservoirs has been one of key strategies underlying successful control in areas with identified zoonotic transmission of *Schistosoma.* This is well documented in China where mammalian hosts, particularly water buffalo, have been shown to play an important role in transmission and human infections of *S. japonicum*, and managing these animal reservoirs (e.g. via drug treatment and/or isolation) has been pivotal in disease control [52]. However, there are very limited studies on *S. mansoni* in potential animal reservoirs in comparison with human infection studies, despite reports of natural infections of the parasite in NHPs. This systematic review indicates that *S. mansoni* infections in African NHPs have been documented across a wide geographical area, and infection rates varied substantially across regions and species/genera (Fig. 2).

**Fig. 6:**
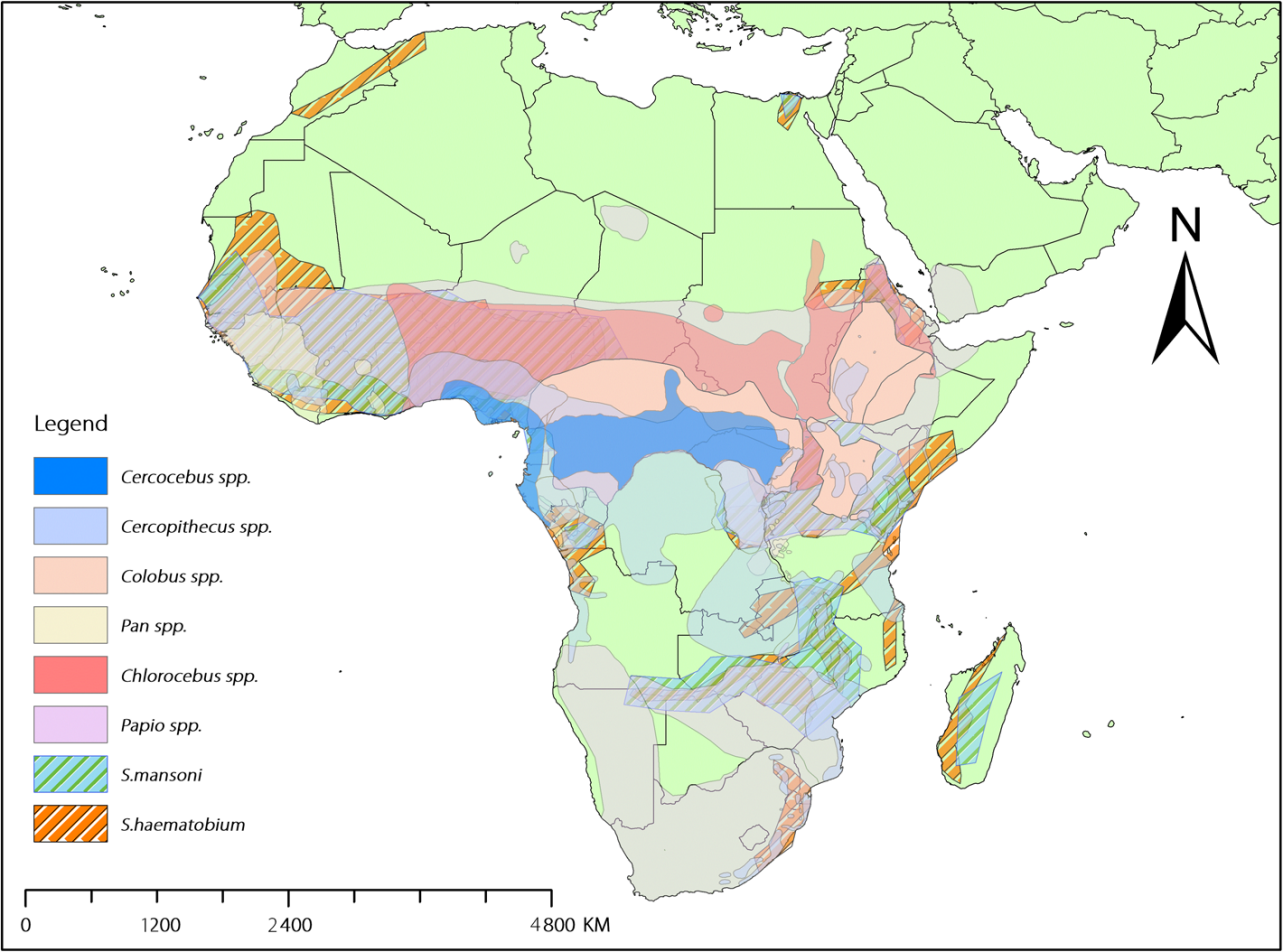
Distribution of endemic areas of *Schistosoma mansoni* and *S. haematobium*, and major genera of nonhuman primates found to be naturally infected by *S. mansoni* in Africa (Data sources: nonhuman primate – https://www.iucnredlist.org; schistosomiasis – http://www.thiswormyworld.org)

We found high heterogeneity in *S. mansoni* infections among African NPHs, and the underlying causes for this are not apparent. However, similarly to other infectious diseases reported in NHPs, the patterns of *S. manosoni* infection are likely mediated by factors associated with the primates themselves, such as group size, social status, habitat use, and behaviour [53, 54]. Kebede et al. reported that *Papio* baboons exhibited patterns of schistosomiasis infections that varied among populations and habitats in Ethiopia [51]. This suggests that within a species, habitat differences may affect infection rates at the subpopulation level. In Africa, NHPs are widely distributed, leading to overlaps with many human *S. mansoni* endemic areas (Fig. 6) and providing potential opportunities for exposure from humans and vice versa*.* Indeed, some early studies in Africa have pointed out that baboons are natural hosts for *S. mansoni* and can maintain transmission in the wild [55, 56]. Given the evidence for baboon-human overlaps and potential parasite sharing [57], a cycle of spillover and spillback maintenance of *S. mansoni* is plausible. Several studies included in this review reported lifecycle experiments of *S. mansoni* from vervet monkeys in which eggs shed by the animals hatched into viable miracidia that infected snails. The infected snails then shed cercariae to infect laboratory mice for subsequent worm harvest, confirming the capacity of the NHPs to maintain local transmission [39, 41].

In this review, the association between NHP and human infections by *S. mansoni* based on available data was also explored. Despite substantial variation and a low sample size, the overall trend was positive. However, such association should be interpreted with a caveat. The information for human and NHP surveys were based on different sampling and diagnostic methods, and certain species of primates might be over-represented (e.g., those most likely to be hunted). However, it is apparent that the overlap of NHP habitats and *S. mansoni* endemic areas has high potential for zoonotic transmission (Fig. 6). Although the epidemiological roles of NHPs in schistosomiasis transmission to humans remain largely unknown, their close interactions in many endemic areas (e.g., sharing water environments that sustain the disease transmission) have raised concerns that the NHPs’ roles in the disease transmission may not be negligible.

To confront the challenge of better understanding this NHP role in the *S. mansoni* transmission, there are knowledge gaps to overcome. Improved understanding of the molecular characteristics of *Schistosoma* strains circulating in humans and NHPs are needed. A few studies described in this review made initial explorations of molecular characterization of the parasites. Bakuza et al. performed a molecular analysis of schistosome eggs from baboons and reported that *S. mansoni* existed in both humans and baboons, but the gene region sequenced was not variable enough to assess whether they shared the same strain [47]. Another study used isoenzyme electrophoresis to compare human and baboon *S. mansoni* strains and found no unique alleles or indication of distinctions between the parasite populations [34]. However, this was not conclusive and more sensitive techniques are needed to confirm the similarity of the parasites from the two types of hosts [34].

Still less well understood are the interactions between the NHPs and humans that could promote zoonotic schistosomiasis transmission. For example, do the humans and NHPs share exposure environments (e.g., water contact and contamination sites)? If so, what are spatial and temporal windows of overlaps? Several studies mentioned crop-raiding as one of interactions with human populations that may influence disease transmission [42, 43]. Another paper mentioned the overlap of ecotourism and NHP habitat at the shores of Lake Langano in Ethiopia and the surrounding forest reserve, which could have implications for transmission through a shared water source [39]. Rigorous quantification of the spatiotemporal overlaps and shared resource use behaviour would greatly improve our understanding of this transmission system.

Nonhuman primate infections by *S. mansoni* not only have potential human public health importance but also notable conservation implications when considering spillover and spillback scenarios. For instance, Cervena et al. [49] surveyed 8 free-ranging Western lowland gorillas (*Gorilla gorilla gorilla*) and found that 5 of them were infected by *S. mansoni*. Gorillas are classified by the International Union for Conservation of Nature (IUCN) as critically endangered [58], and are highly susceptible to spillover infections from humans [59]. Do *S. mansoni* infections pose health threats to the gorillas? If so, what are the associated morbidities? Other primate species identified in this review are also of conservation concern: *Pan troglodytes* is endangered and *Papio papio* is classified as near-threatened [60, 61]. Further knowledge on *S. mansoni* infections of NHPs and its health impacts, if any, may provide important information for the management and preservation of wildlife populations. This would also clarify roles of NHP species as definitive, maintenance (impacted or not), or spillover hosts.

Our meta-analysis was limited to studies with directly comparable information. Primate genera which only appeared in single studies were excluded from the meta-analysis part of this review. The study had several additional limitations. First, although studies with sample size < 10 were excluded from the meta-analysis, some included studies still had modest sample sizes and therefore may not adequately represent population level infection rates. Sample sizes in the studies varied considerably, from 3 to 206, with different sampling strategies, so pooled estimates were likely subject to impacts of varying sampling schemes. Second, the studies used 12 different diagnostic techniques for infection with FECT, Kato-Katz thick smear, and necropsy being the most commonly used. These tests have different levels sensitivity and specificity in the diagnosis of schistosome presence, which we did not take into account explicitly, and which may introduce bias in the calculation of pooled estimates. Nevertheless, such estimates represent a conservative quantification of actual infections, and to some extent, serve the purpose of this review.

## Conclusions

There is substantial overlap of habitats of nonhuman primate (NHP) species and schistosomiasis endemic areas in Africa. Our review suggests that the reported *S. mansoni* infection rates in NHPs are generally high, with substantial variations across geographical regions and primate species/genera. There is a pressing need for an improved understanding about the potential zoonotic roles of these NHPs in the transmission cycle of human schistosomiasis.

## Additional file

Additional file 1: Multilingual abstracts in the five official working languages of the United Nations. (PDF 504 kb)

## Abbreviations

CCA: Circulating cathodic antigen test
CI: Confidence interval
ELISA: Enzyme-linked immunosorbent assay
FECT: Formal-ether concentration technique
IUCN: International Union for Conservation of Nature
MBT: Modified Baerman’s technique
MIF: Merthiolate-iodine-formalin fecal technique
MMT: Modified McMaster technique
MSF: Modified Sheather’s flotation
NHP: Non-human primate
NTD: Neglected tropical disease
qPCR: Real-time polymerase chain reaction
SNF: Sodium nitrate flotation
WHO: World Health Organization
ZSF: Zinc sulfate flotation
