# Supplementary material for: Assessing the nonhuman primate reservoir of Schistosoma mansoni in Africa: a systematic review
Source: Infect Dis Poverty. 2019 May 10;8:32. doi: 10.1186/s40249-019-0543-7 (PMC6509776; doi:10.1186/s40249-019-0543-7)
Supplement: Supplementary file 1 — Multilingual abstracts in the five official working languages of the United Nations. (PDF 504 kb) [file 40249_2019_543_MOESM1_ESM.pdf]

Translation of the abstract into the five official working languages of the United Nations

تقييم الخزان الرئيسى غير البشري للبلهارسيا المانسونيةفي أفريقيا: مراجعة منهجية

ليندساي ريتشاردز ، برهانو إركو ، كيراتي بونبيتش ، سادي ج رايان وسونج ليانج

الملخص

الخلفية: إن تقارير الإصابات الطبيعية بالبلهارسيا المانسونية في عدد من أنواع الرئيسيات غير البشرية (NHPs) في أفريقيا ، إلى جانب التداخل الكبير لبيئات NHP والمناطق التي تستوطنها البلهارسيا البشرية ، قد أدت إلى مخاوف بشأن دور الرئيسيات غير البشرية NHPs في انتقال البلهارسيا البشرية. أجرينا مراجعة منهجية للمطبوعات لوصف نطاق المعرفة الحالي بالنسبة لأفريقيا ، لأنواع الرئيسيات غير البشرية NHP المتورطة ، وتوزيعها الجغرافي ، ومعدلات الإصابة بالبلهارسيا المانسونية , ومناقشة الآثار المترتبة على الصحة العامة والحفظ .

المتن الرئيسي: تم إجراء بحث منهجي في المطبوعات باستخدام PubMed و Web of Science و Google Scholar وقاعدة بيانات مكتبة منظمة الصحة العالمية (WHO) و World Cat و ScienceDirect دون أي قيود لغوية. وأُدرجت الدراسات التي تفحص الإصابة بمرض البلهارسيا المانسونية لأي نوع من أنواع الرئيسيات غير البشرية NHP الإفريقية. تم التلخيص النوعي لأنماط الدراسة وأنواع الرئيسيات وتوزيعها الجغرافي وتقنيات تشخيص الطفيليات الواردة في الدراسات. تم تضمين بيانات الأنواع ذات أحجام العينات ≤ 10 في التحليل التلوي. قمنا بتقييم معدل الإصابة المبلغ عنه ، واستخدمنا نموذجاً للتأثيرات العشوائية لتقدير معدلات الإصابة الموجزة وفواصل الثقة 95٪ (CIs). وقمنا بتقييم عدم التجانس بين الدراسات باستخدام إحصائيات I^2 . تم تحديد تسعة وعشرين منشوراً ، من 1960 إلى 2018 ، وأدرجت في المراجعة. فحصت الدراسات إجمالي 2962 من الرئيسيات ينتمون إلى 22 نوعاً في 11 جنساً عبر 10 دول (الكاميرون وإريتريا وإثيوبيا والجابون وكينيا ونيجيريا والسنغال وتنزانيا وأوغندا وزيمبابوي) ، وتم العثور على إصابات بلهارسيا مانسونية في تسعة أنواع من خمسة أجناس في جميع البلدان. عندما استبعدنا الدراسات ذات أحجام العينة <10 ، بقيت البيانات من 24 دراسة حول 11 نوعاً من الرئيسيات في ثلاثة أجناس في عشرة بلدان في التحليل التلوي. كان التقدير المجمع الإجمالي لمعدل الإصابة 10٪ (95% CI: 6–16%) مع عدم تجانس عالي (I^2 = 94.77%) عبر البلدان والأنواع/الأجناس. من بين الأجناس الثلاثة ، كان Pan أعلى معدل إصابة بنسبة 15٪ (95% CI: 0–55%)، متبوعة Papio بنسبة 11٪ (95% CI: 6–18%) و Cercopithecus بنسبة 5٪ (95% CI: 0–14%). كانت العلاقة بين الرئيسيات غير البشرية NHP والإصابات البشرية إيجابية ، ولكن ليست كبيرة ، بسبب انخفاض تطابقات عينة الدراسة والتباين العالي.

الاستنتاجات: تشير نتائجنا إلى أن معدل الإصابة بالبلهارسيا المانسونية مرتفع في أنواع الرئيسيات غير البشرية NHPs الأفريقية ، مع وجود تباينات كبيرة بين الأنواع / الأجناس والبلدان في أفريقيا. نظراً للأدلة التي تشير إلى احتمال حدوث تداعيات وامتداد للبلهارسيا المانسونية بين أنواع الرئيسيات غير البشرية NHPs الأفريقية والبشر ، هناك حاجة ماسة إلى مزيد من البحث لفهم البيئة وآليات انتقال الطفيل بين الرئيسيات غير البشرية NHP والمضيفات البشرية ، من أجل إبلاغ استراتيجيات السيطرة على هذا المرض المداري المهمل.

Translated from English version into Arabic by Aalya Al-Beeshi, Revised by Shaima'a al-Fahel, through

TRANSLATORS
WITHOUT BORDERS

非洲曼氏血吸虫灵长类动物传染源的系统综述

Lindsay Richards, Berhanu Erko, Keerati Ponpetch, Sadie J. Ryan and Song Liang

摘要

引言： 在非洲，灵长类多种动物时有自然感染曼氏血吸虫的报道。这些动物的栖息地和血吸虫病流行区有很大幅度的重叠，不禁引发人们对这些灵长类动物是否在血吸虫病传播中起作用的关注。我们进行了系统的文献综述，总结目前所知的情况，包括涉及到的灵长类动物种类，它们的地理分布，感染曼氏血吸虫的情况，并讨论了公共卫生及资源保护的意义。

正文：文献检索利用了如下数据库，PubMed, Web of Science, Google Scholar，世界卫生组织图书馆数据库, World Cat，及 ScienceDirect。检索没有语言限制。综述包括了非洲灵长类动物自然感染曼氏血吸虫的研究报告，总结了有关报告类型，涉及的灵长类动物种属，地理分布，及诊断方法。调查样本量≥10 的报告数据纳入 Meta 分析。定量分析利用报告的感染率，用随机效应模型（random-effect model）估算基于报告国家及灵长类属为单位的汇总感染率及 95%可信区间。报告的异质性采用 I^2 统计量。共 29 篇发表于 1960 至 2018 间的报告符合条件被纳入综述。报告包括总计 2962 只灵长类动物，涵盖 22 种 11 个属，分布于 10 个国家（喀麦隆，厄立特里亚，埃塞俄比亚，加蓬，肯利亚，尼日利亚，塞内加尔，坦桑尼亚，乌干达，及津巴布韦）。这些国家中共有 5 属 9 种灵长类动物有感染曼氏血吸虫的报道。 排除样本量小于 10 的报告，共有涉及所有国家 3 属 11 种灵长类动物的 24 篇文章的数据被纳入 meta 分析。报告的感染率在跨国及跨种属间有显著的异质性（I^2 = 94.77%)，汇总估计的感染率为 10%(95% CI: 6-16%)。在这三属中，黑猩猩属(*Pan*) 的感染率最高，达 15%（95% CI：0-55%），其次为狒狒属(*Papio*)感染率为 11%（95% CI：6-18%），最后为长尾猴属（*Cercopithecus*）感染率为 5%（95% CI：0-14%）。灵长类动物的感染率与对应地区的人群感染总趋势呈正相关，但由于较低的研究报告匹配数量及较高的报告结果差异，该关系在统计上不显著。

结论：非洲灵长类动物曼氏血吸虫自然感染率高，在不同国家及种属间差异很大。据现有证据显示在非洲灵长类动物和人之间曼氏血吸虫可能的互相传播，对曼氏血吸虫在这两类宿主间传播的生态学和机制的进一步研究刻不容缓，以指导对这一重要的被忽略的热带病控制。

Translated from English version into Chinese by Song Liang

Évaluation du réservoir de *Schistosoma mansoni* parmi les primates non humains en Afrique : revue systématique

Lindsay Richards, Berhanu Erko, Keerati Ponpetch, Sadie J. Ryan et Song Liang

Résumé

Contexte : des rapports de cas d'infestation naturelle par *Schistosoma mansoni* de plusieurs espèces africaines de primates non humains (PNH), sachant que l'habitat des PNH recoupe largement les zones de schistosomiase humaine endémique, suscitent des inquiétudes quant au rôle des PNH dans la transmission de la schistosomiase humaine. Nous avons mené une revue systématique de la littérature afin de faire le point des connaissances actuelles sur les espèces de primates impliquées, leur distribution géographique et le taux d'infestation par *S. mansoni* dans le contexte africain

ainsi que de discuter des implications de ces données pour la santé publique et la protection des espèces.

Discussion : nous avons mené une recherche systématique dans la littérature via PubMed, Web of Science, Google Scholar, la base de données de littérature de l'Organisation mondiale de la santé (OMS), World Cat et ScienceDirect, sans restriction en termes de langue. Nous avons inclus les études examinant l'infestation par *S. mansoni* de quelconques espèces africaines de PNH. Le type des études, les espèces de primates, leur distribution géographique et les techniques de diagnostic parasitaire rapportés dans les études ont été résumés qualitativement. Nous avons inclus à la méta-analyse les données relatives aux espèces pour lesquelles la taille d'échantillon était ≥ 10. Nous avons évalué le taux d'infestation et utilisé un modèle à effets aléatoires pour estimer les taux d'infestation totaux et les intervalles de confiance (*IC*) à 95 %. Nous avons évalué l'hétérogénéité entre les études à l'aide des statistiques de I^2. Nous avons identifié et inclus à la revue 29 publications parues entre 1960 et 2018. Les études portaient sur un total de 2962 primates appartenant à 22 espèces de 11 genres dans 10 pays (Cameroun, Érythrée, Éthiopie, Gabon, Kenya, Nigeria, Ouganda, Sénégal, Tanzanie et Zimbabwe) et une infestation par *S. mansoni* a été observée chez 9 espèces de 5 genres dans tous ces pays. Après exclusion des études dont la taille d'échantillon était inférieure à 10, il nous est resté des données de 24 études sur 11 espèces de primates de 3 genres, dans 10 pays. L'estimation regroupée globale du taux d'infestation était de 10 % (*IC* à 95 % de 6 à 16 %), avec une grande hétérogénéité (I^2 = 94,77 %) entre les pays et entre les espèces et genres. Parmi les trois genres, c'est *Pan* qui présentait le taux d'infestation le plus élevé avec 15 % (*IC* à 95 % de 0 à 55 %), suivi de *Papio* avec 11 % (*IC* à 95 % de 6 à 18 %) et *Cercopithecus* avec 5 % (*IC* à 95 % de 0 à 14 %). L'association entre les PNH et les infestations humaines était positive, mais non significative en raison des correspondances réduites entre les échantillons des études et de leurs fortes variations.

Conclusions : nos résultats suggèrent que le taux d'infestation par *S. mansoni* est élevé parmi les PNH africains, avec des hétérogénéités significatives entre les espèces et genres et entre les pays d'Afrique. Dans la mesure où les données suggèrent une transmission dans les deux sens (« spillover » et « spillback ») de *S. mansoni* entre les PNH africains et les humains, de nouvelles recherches sont nécessaires d'urgence pour comprendre l'écologie et les mécanismes de transmission du parasite entre les hôtes PNH et humains, afin de fournir des informations à l'appui des stratégies de lutte contre cette importante maladie tropicale négligée.

Translated from English version into French by Suzanne Assenat, Revised by Eric Ragu, through

TRANSLATORS
WITHOUT BORDERS

Оценка естественного резервуара *Schistosoma mansoni* у приматов, не являющихся людьми, в Африке: систематический обзор

Линдси Ричардс, Берхану Эрко, Кирати Понпетч, Сэди Дж. Райан и Сонг Лян

Аннотация

Общая информация: сообщения о естественных инфекциях *Schistosoma mansoni* у ряда видов приматов, не являющихся людьми, (ПНЯЛ) в Африке в сочетании с существенным перекрытием мест обитания ПНЯЛ и эндемичных районов шистосомоза человека вызвали озабоченность по поводу роли ПНЯЛ в передаче человеческого шистосомоза. Мы провели систематический обзор литературы с целью описания нынешнего объема знаний для Африки, соответствующих видов ПНЯЛ, их географического распределения, показателей инфицирования *S. mansoni*, а также для обсуждения последствий для общественного здравоохранения и охраны природы.

Основная часть: систематический поиск литературы осуществлялся с использованием PubMed, Web of Science, Google Scholar, библиотечной базы данных Всемирной организации здравоохранения (ВОЗ), World Cat и ScienceDirect без каких-либо языковых ограничений. Были включены исследования, изучающие инфицирование *S. mansoni* любого вида африканских ПНЯЛ. Были качественно обобщены типы исследований, виды приматов, их географическое распределение, методы диагностики паразитов, представленные в исследованиях. В мета-анализ были включены данные по видам с размерами выборки ≥ 10. Мы оценили сообщенную частоту инфицирования и использовали модель случайных эффектов для оценки суммарных показателей инфицирования и 95% доверительных интервалов (*ДИ*). Мы оценили неоднородность исследований с использованием статистики I^2. В период с 1960 по 2018 год было выявлено и включено в обзор 29 публикаций. В ходе исследований было изучено в общей сложности 2962 примата, принадлежащих к 22 видам в 11 родах в десяти странах (Габон, Зимбабве, Камерун, Кения, Нигерия, Сенегал, Танзания, Уганда, Эритрея и Эфиопия), а инфекции *S. mansoni* были обнаружены у девяти видов из пяти родов во всех странах. Когда мы исключили исследования с размерами выборки < 10, то в метаанализе остались данные 24 исследований по 11 видам приматов из трех родов в десяти странах. Общая объединенная оценка уровня инфекции составила 10 % (95 % *ДИ*: 6-16 %) с высокой гетерогенностью (I^2 = 94,77 %) по странам и видам/родам. Среди трех родов, у *Pan* был самый высокий уровень заболеваемости — 15 % (95 % *ДИ*: 0-55 %), затем *Papio* — 11 % (95 % *ДИ*: 6-18 %) и *Cercopithecus* — 5 % (95 % *ДИ*: 0-14 %). Связь между ПНЯЛ и человеческими инфекциями была положительной, но не значительной, из-за низких совпадений исследуемых выборок и высокой вариативности.

Выводы: наши результаты показывают, что уровень инфицирования *S. mansoni* высок в африканских ПНЯЛ, с существенными неоднородностями по видам/родам и странам Африки. С учетом фактических данных о потенциальном распространении и обратном распространении *S. mansoni* между африканскими ПНЯЛ и людьми срочно необходимы дальнейшие исследования для понимания экологии и механизмов передачи паразита между хозяевами-ПНЯЛ и хозяевами-людьми, с тем чтобы собрать информацию для разработки стратегии борьбы с этим важным, но оставленным без внимания тропическим заболеванием.

Translated from English version into Russian by Michael Orlov, Revised by Alexander Somin, through

TRANSLATORS
WITHOUT BORDERS

Evaluación del depósito de *Schistosoma mansoni* en primates no humanos en Africa: una revisión sistemática

Lindsay Richards, Berhanu Erko, Keerati Ponpetch, Sadie J. Ryan y Song Liang

Resumen

Antecedentes: los informes de infecciones naturales de *Schistosoma mansoni* en varias especies de primates no humanos (NHP, por sus siglas en inglés) en África, junto con la superposición sustancial de los hábitats de NHP y las áreas endémicas de esquistosomiasis humana, han generado preocupación sobre el papel de los NHP en la transmisión de la esquistosomiasis humana. Llevamos a cabo una revisión sistemática de la literatura para describir el alcance actual del conocimiento para África, para las especies de NHP implicadas, su distribución geográfica, las tasas de infección con *S. mansoni*, y para discutir las implicaciones para la salud pública y la conservación.

Texto principal: se realizó una búsqueda sistemática de literatura utilizando PubMed, Web of Science, Google Scholar, la base de datos de la biblioteca de la Organización Mundial de la Salud (OMS), World Cat y ScienceDirect, sin ninguna restricción de idioma. Se incluyeron los estudios de la infección por *S. mansoni* de todas las especies de NHP africanas. Los tipos de estudios, las especies de primates, su distribución geográfica y las técnicas de diagnóstico de parásitos informadas en los estudios se resumieron cualitativamente. Los datos para las especies con tamaños de muestra ≥ 10 se incluyeron en el metanálisis. Evaluamos la tasa de infección informada y utilizamos un modelo de efectos aleatorios para estimar las tasas de infección resumidas y los intervalos de confianza del 95 % (*CI*, por sus siglas en inglés). Se evaluó la heterogeneidad entre los estudios utilizando la estadística I^2. Veintinueve publicaciones, de 1960 a 2018, fueron identificadas e incluidas en la revisión. Los estudios examinaron un total de 2962 primates pertenecientes a 22 especies de 11 géneros en diez países (Camerún, Eritrea, Etiopía, Gabón, Kenia, Nigeria, Senegal, Tanzania, Uganda y Zimbabwe) y se encontraron infecciones por *S. mansoni* en nueve especies de cinco géneros en todos los países. Cuando se excluyeron los estudios con tamaños de muestra < 10, los datos de 24 estudios sobre 11 especies de primates de tres géneros en diez países permanecieron en el metanálisis. La estimación global combinada de la tasa de infección fue del 10 % (CI del 95 %: 6–16 %) con alta heterogeneidad (I^2 = 94,77 %) en todos los países y especies / géneros. En los tres géneros, *Pan* tuvo el más alto índice de infección del 15 % (CI del 95 %: 0–55 %), seguido por *Papio* con un 11 % (CI del 95 %: 6–18 %) y *Cercopithecus* con un 5 % (CI del 95 %: 0–14 %). La asociación entre los NHP y las infecciones en humanos fue positiva, pero no significativa, debido a la baja correspondencia de las muestras de estudio y la gran variación.

Conclusiones: nuestros hallazgos sugieren que el índice de infección por *S. mansoni* es alto en los NHP de África, con una heterogeneidad considerable entre especies/géneros y países del continente. Dadas las pruebas de posibles contagios y de reinfección por *S. mansoni* entre los NHP africanos y los humanos, se necesita urgentemente más investigación para comprender la ecología y los mecanismos de transmisión del parásito entre los NHP y los huéspedes humanos, a fin de informar las estrategias de control de esta importante enfermedad tropical desatendida.

Translated from English version into Spanish by Mayra León, Revised by Patricia Cassoni, through

TRANSLATORS
WITHOUT BORDERS
